# Sloths host Anhanga virus‐related phleboviruses across large distances in time and space

**DOI:** 10.1111/tbed.13333

**Published:** 2019-09-05

**Authors:** Edmilson F. de Oliveira Filho, Andrés Moreira‐Soto, Carlo Fischer, Andrea Rasche, Anna‐Lena Sander, Judy Avey‐Arroyo, Francisco Arroyo‐Murillo, Eugenia Corrales‐Aguilar, Jan Felix Drexler

**Affiliations:** ^1^ Institute of Virology Charité‐Universitätsmedizin Berlin corporate member of Freie Universität Berlin Humboldt‐Universität zu Berlin Berlin Institute of Health Berlin Germany; ^2^ Faculty of Microbiology, Virology‐CIET (Research Center for Tropical Diseases) University of Costa Rica San José Costa Rica; ^3^ The Sloth Sanctuary of Costa Rica Limon Costa Rica; ^4^ German Centre for Infection Research (DZIF), associated partner site Charité Berlin Germany; ^5^ Martsinovsky Institute of Medical Parasitology, Tropical and Vector-Borne Diseases Sechenov University Moscow Russia

**Keywords:** arbovirus, Costa Rica, evolution, penshurt virus, phlebovirus, sloths

## Abstract

Sloths are genetically and physiologically divergent mammals. Phleboviruses are major arthropod‐borne viruses (arboviruses) causing disease in humans and other animals globally. Sloths host arboviruses, but virus detections are scarce. A phlebovirus termed Anhanga virus (ANHV) was isolated from a Brazilian Linnaeus's two‐toed sloth (*Choloepus didactylus*) in 1962. Here, we investigated the presence of phleboviruses in sera sampled in 2014 from 74 Hoffmann's two‐toed (*Choloepus hoffmanni*, *n* = 65) and three‐toed (*Bradypus variegatus*, *n* = 9) sloths in Costa Rica by broadly reactive RT‐PCR. A clinically healthy adult Hoffmann's two‐toed sloth was infected with a phlebovirus. Viral load in this animal was high at 8.5 × 10^7^ RNA copies/ml. The full coding sequence of the virus was determined by deep sequencing. Phylogenetic analyses and sequence distance comparisons revealed that the new sloth virus, likely representing a new phlebovirus species, provisionally named Penshurt virus (PEHV), was most closely related to ANHV, with amino acid identities of 93.1%, 84.6%, 94.7% and 89.0% in the translated L, M, N and NSs genes, respectively. Significantly more non‐synonymous mutations relative to ANHV occurred in the M gene encoding the viral glycoproteins and in the NSs gene encoding a putative interferon antagonist compared to L and N genes. This was compatible with viral adaptation to different sloth species and with micro‐evolutionary processes associated with immune evasion during the genealogy of sloth‐associated phleboviruses. However, gene‐wide mean dN/dS ratios were low at 0.02–0.15 and no sites showed significant evidence for positive selection, pointing to comparable selection pressures within sloth‐associated viruses and genetically related phleboviruses infecting hosts other than sloths. The detection of a new phlebovirus closely‐related to ANHV, in sloths from Costa Rica fifty years after and more than 3,000 km away from the isolation of ANHV confirmed the host associations of ANHV‐related phleboviruses with the two extant species of two‐toed sloths.

## 1 INTRODUCTION

Sloths belong to the Xenarthra superorder, whose members are ancient placental mammals that are extant only in the Americas (de Moraes‐Barros, Silva, Miyaki, & Morgante, 2006). Phleboviruses are ubiquitous arthropod‐borne viruses (arboviruses), associated with human and animal disease, such as Rift Valley fever virus (RVFV), Punta Toro virus (PTV), Toscana virus (TOSV), Sandfly Naples virus (SFNV) and severe fever with thrombocytopenia syndrome virus (SFTSV) (Ayhan & Charrel, 2017; Gundacker et al., 2017; Hartman, 2017). The phleboviral genome consists of three single‐stranded RNA segments of negative or ambisense polarity, termed the large (L), medium (M) and small (S) segments. The L segment encodes the RNA‐dependent RNA polymerase (RdRp), and the M segment encodes a polyprotein which is cleaved into a non‐structural protein (NSm) and two envelope glycoproteins termed Gn and Gc. Finally, the S segment encodes the nucleocapsid (N) transcribed in the viral sense and a non‐structural protein (NSs) transcribed in reverse sense (Hornak, Lanchy, & Lodmell, 2016).

Sloths are understudied regarding the viruses they host. Serological studies suggest circulation of different arboviruses belonging to the *Alphavirus*, *Flavivirus*, *Orthobunyavirus* and *Phlebovirus* genera in different species from both extant sloth genera *Bradypus* and *Choloepus* in Central and South America (Gilmore, Da Costa, & Duarte, 2001; Medlin et al., 2016; Seymour, Peralta, & Montgomery, 1983a; de Thoisy, Dussart, & Kazanji, 2004; de Thoisy, Gardon, Salas, Morvan, & Kazanji, 2003). However, cross‐reactivity of antibodies between antigenically related arboviruses precludes definite assessments of the arboviruses infecting sloths. Viruses that have been genetically characterized from sloths include, among others, the orthobunyavirus Oropouche virus (OROV), the orbivirus Changuinola virus (CHV) and the two phleboviruses PTV and Anhanga virus (ANVH) (Seymour et al., 1983a; Seymour, Peralta, & Montgomery, 11983b;; Travassos da Rosa et al., 1984, 2017). For comparison, genomic sequences of coronaviruses alone mount to several hundreds to thousands in other mammalian orders such as bats, carnivores or ungulates, representing multiple viral species and genera (Drexler, Corman, & Drosten, 2014), thus highlighting the scarcity of viral genomic information available from sloth hosts.

ANHV was isolated from a single Linnaeus's two‐toed sloth (*Choloepus didactylus*) from Brazil in 1962, and its complete genomic sequence has only recently become available (Nunes‐Neto et al., 2017; Tesh, 1988). After 1962, no subsequent detection in wildlife or humans has been documented, which limits studies on the distribution and diversity of this virus and on the potential role of sloths as hosts of ANHV. Here, we characterize a new ANHV‐related phlebovirus species in sloths from Costa Rica.

## 2 MATERIALS AND METHODS

**Figure 1:**
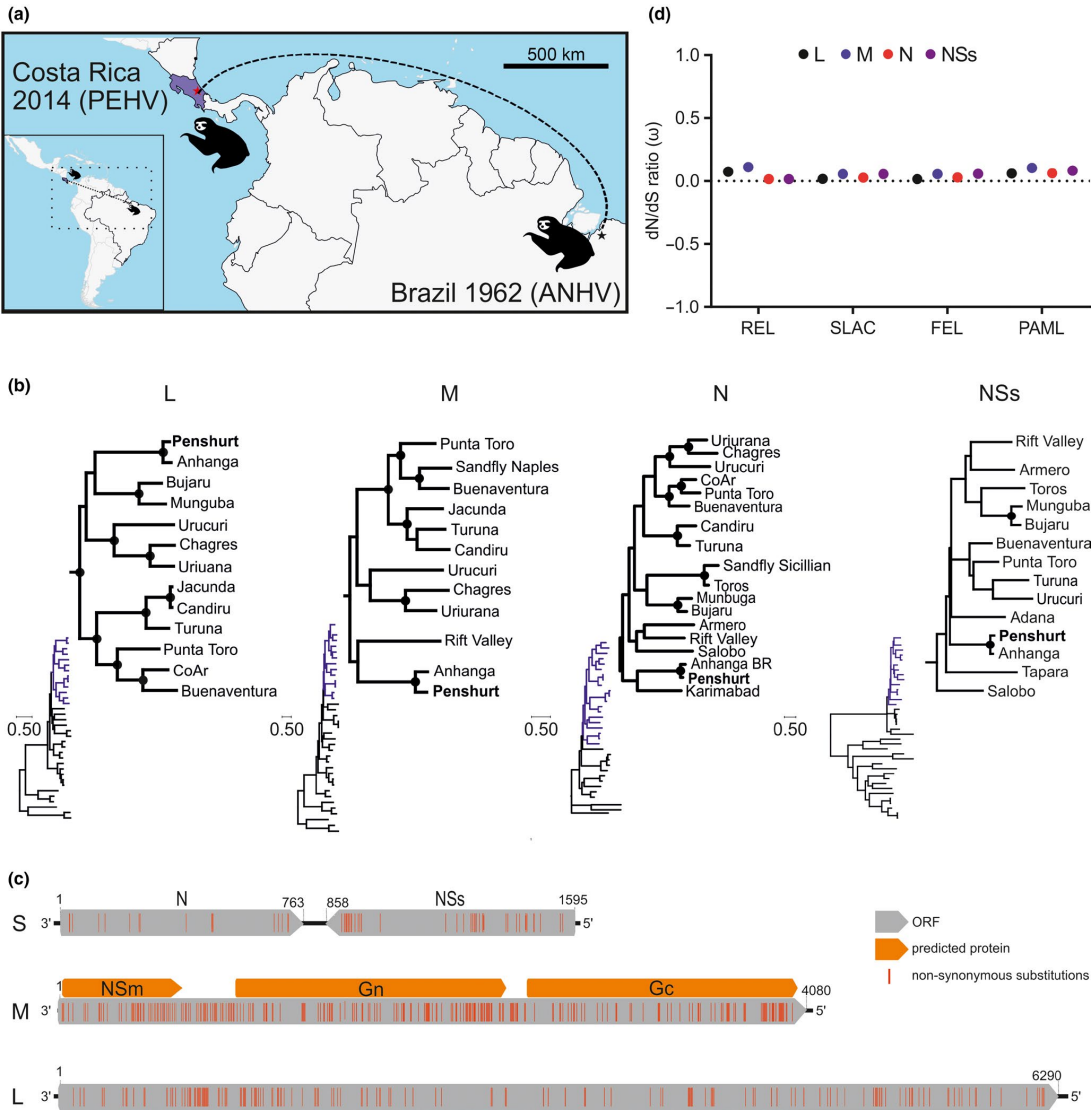
Location, genomic and evolutionary characteristics of the novel phlebovirus found in a Costa Rican sloth. Geographic distribution of ANHV‐related viruses, that is, ANHV from Brazil and Penshurt virus (PEHV) from Costa Rica. Map built with Quantum GIS—QGIS (http://qgis.osgeo.org) (a). Maximum likelihood phylogenetic tree based on translated amino acid sequences of the L, M, N and NSs genes with highlighted trees showing viruses closely related to ANHV and PEHV. Black circles at nodes represent support values of ≥0.75 from 1000 bootstrap replicates (b). Genome organization of the new PEHV found in Costa Rican sloths, showing genomic segments and encoded proteins (RdRp on L; NSm, Gn and Gc on M; nucleocapsid (N) and non‐structural (NSs) on S segment). Proteins were predicted according to BLASTp analysis comparing the PEHV genome with those from other pheboviruses. Red, non‐synonymous substitutions compared to ANHV reference sequences (c). Mean dN/dS (ω) ratios on L, M, N and NSs genes using different methods as indicated below (d). Pressure analyses included the two ANHV‐related strains ANHV and PEHV and Bujaru, Candiru, Chagres, CoAr, Munguba, Punta Toro, Rift Valley, Salobo, Turuna and Uriana virus (d). Accession number for all phlebovirus reference sequences (segments L, M and S) used: Adana (KJ939330, NC_029128, NC_029129), Anhanga (NC_033836, NC_033846, NC_033837), Armero (HM566140, HM566141, HQ661807) Bujaru (KX611388, KX611389, KX611390), Buenaventura (KP272001, KP272002, EF201839), Candiru (NC_015374, NC_015373, NC_015375), Chagres (HM566147, HM566146, HM566148), CoAr (HM566152, HM566154, HM566153), Jacunda (HM466934, HM466935, HM466936), Karimabad (KF297912, KF297913, KF297914), Munguba (HM566164, NC_033847, NC_033831), Punta Toro (KP272004, KP272005, KP272006), Rift Valley (DQ375409, DQ380204, DQ380146), Salobo (HM627185, HM627183, HM627184), Sandfly Naples (HM566172, HM566169, HM566170), Sandfly Sicilian (NC_015412, NC_015411, NC_015413), Palma (NC_021242, NC_021243, NC_021244), Toros (KP966619, NC_037614, NC_037615), Turuna (HM119431, HM119432, HM119433), Uriurana (HM566189, HM566188, HM566190), Urucuri (NC_033841, NC_033842, NC_033843) [Colour figure can be viewed at http://www.wileyonlinelibrary.com/]

A total of 74 sera were sampled from 65 individual Hoffmann's two‐toed (*Choloepus hoffmanni*) and 9 three‐toed (*Bradypus variegatus*) sloths by venous puncture done by trained veterinarians on the ‘Sloth Sanctuary’ in Costa Rica in 2014 (http://www.slothsanctuary.com; Figure 1a). Of the 74 sloths, 70 were captive, three had recently arrived and were in quarantine, and one was a wild sloth, which had entered the sanctuary. Permission for sampling was obtained from the National Council in the Management of Biodiversity (resolution R‐026‐OT‐CONAGEBIO) according to international animal health standards.

Viral RNA was extracted using the RNeasy Kit (Qiagen). Samples were screened for phlebovirus RNA using a broadly reactive RT‐PCR assay (Lambert & Lanciotti, 2009). Because broadly reactive assays may be limited in sensitivity due to nucleotide mismatches below oligonucleotide primers (Drexler et al., 2007), a strain‐specific hemi‐nested screening RT‐PCR assay was designed subsequent to genomic characterizations to augment sensitivity (Table 1). Viral RNA was quantified using a strain‐specific real‐time RT‐PCR assay (Table 1) controlled by photometrically quantified in vitro‐transcribed RNA controls generated as described previously (Drexler et al., 2009). Deep sequencing was done using MiSeq Reagent v2 chemistry (Illumina), the KAPA Frag Kit and a KAPA RNA HyperPrep library (Roche). Sequence assembly and MAFFT alignments were done in Geneious 9.1.8 (http://www.geneious.com).

**Table 1:** Primers for detection and quantification of PEHV^a^

Use	Gene	Name	Oligonucleotide sequence
RT‐PCR	L	L69F	CTTAGCTATGTTGAAACCCTATACA
		L409R	GCCAATAGGGCAGATTTCTCTG
		L394R	TCTCTGCTCCTCTAGTTGTGG
qRT‐PCR	L	PEHV‐rtF	AAGATTGTGAGAATGTGCACTGAGT
		PEHV‐rtR	TTAGTGTCCGCTCCCTTTCCT
		PEHV‐rtP	TTGGAACAGTATTCTCACACTTGCCGGCT^b^

a Thermocycling involved reverse transcription at 55°C for 20 min followed by 94°C for 3 min and then 45 cycles of 94°C for 15 s and 58°C for 30 s. PCR was done using the One‐Step SuperScript III RT‐PCR kit for first‐round and real‐time RT‐PCR reactions and the Platinum *Taq* PCR kit (Thermo Fisher) for second round reactions.

b Labelled with fluorescein amidite (FAM) at the 5′ end and a dark quencher at the 3′ end.

Maximum likelihood (ML) phylogenies were done on translated genes or genomic domains using MEGA X (Kumar, Stecher, Li, Knyaz, & Tamura, 2018) with a WAG amino acid substitution model, 1000 bootstrap replicates and a complete deletion of all alignment positions containing gaps. Recombination was assessed using the Recombination Detection Package V4 (Martin et al., 2010) and GARD (Kosakovsky Pond, Posada, Gravenor, Woelk, & Frost, 2006). Analyses of selection pressure were done using fixed‐effects likelihood (FEL) and single‐likelihood ancestor counting (SLAC), both using the GTRxMG94 substitution model as done in Lam et al. (2013), and random‐effects likelihood (REL; substitution model HKY85 as done in Feng et al. (2019) within Datamonkey from the HYPHY package (Pond & Frost, 2005) and comparing the M1 versus M2 and M7 versus M8 models implemented in the CodeML program in the PAML package using the codon frequency model F61 (Xu & Yang, 2013). Since formal selection pressure analyses within ANHV‐related viruses are limited by the small data set, we selected the 10 genetically most closely related phleboviruses based on amino acid pairwise sequence distances within the complete L gene for these analyses (details on those viruses are provided in the figure legend). Chi‐square tests comparing the occurrence of non‐synonymous mutations between ANHV‐related virus genes and 95% confidence intervals were inferred using Prism version 6.01 (GraphPad Software, http://www.graphpad.com).

The conditions used during the original ANHV isolation are unclear. The virus can be propagated on Vero cells (Nunes‐Neto et al., 2017), yet the initial isolation may have been performed by intracerebral inoculation in suckling mice according to the American Type Culture Collection product entry (ATCC VR‐424). Here, virus isolation was attempted from sloth serum diluted at 1:10 and 1:100 and inoculated onto Vero E6 maintained at 37°C and cultivated in DMEM, and C6/36 cells maintained at 28°C and cultivated in Leibovitz's L15 medium. After 1‐hr incubation, the inoculum was removed and replaced by medium supplemented with 5% foetal calf serum (FCS), 1% penicillin/streptomycin (20 U/ml) and 1% non‐essential amino acids. Infected cells were passaged three times every 7 days and controlled daily for cytopathic effect (CPE). In addition, a quantitative real‐time RT‐PCR assay was used to analyse potential isolation without CPE in supernatant from each passage (Table 1). After three passages, neither CPE nor an increment of RNA levels were observed, potentially due to degradation of the inoculum due to sampling in tropical conditions and repeated freeze‐thawing cycles. GenBank accession numbers for the ANHV‐related phlebovirus, Penshurt virus (PEHV) described here are MN163121 (L segment), MN163122 (M segment) and MN163123 (S segment).

## 3 RESULTS

Both the broadly reactive screening assay and the strain‐specific RT‐PCR assay yielded only one positive sample out of 74 (1.4%; 95% confidence interval, 0.03–7.3). Viral load in that serum was high at 8.5 × 10^7^ RNA copies/ml. The positive animal was a wild and clinically healthy adult Hoffmann's two‐toed sloth, whereas all captive sloths were PCR‐negative. Unfortunately, the exact time of entry of the wild animal into the sanctuary was not known, and whether the animal acquired phlebovirus infection in the wild or in the sanctuary remained unclear. The full coding sequence of the viral genome was recovered by deep sequencing. Comparison of the tree topology based on the four deduced proteins showed that the new sloth virus consistently clustered within the clade of mosquito/sandfly‐associated phleboviruses together with the ANHV prototype strain isolated in Brazil in 1962 (Nunes‐Neto et al., 2017) (Figure 1b). The four amino acid‐based ML trees displayed distinct clustering patterns; while in the L segment the two ANHV‐related strains were most related to Bujaru and Munguba viruses, the ANHV clade did not cluster in close relationship to other phleboviruses in trees based on the M, N and NSs segments (Figure 1b). No reassortment of the ANHV‐related viruses with other phleboviruses and no recombination events within ANHV‐related genes were detected.

The genome of the new ANHV‐related virus encompassed, as observed in other phleboviruses, two negative‐sense segments and an ambisense genomic segment, the coding sequences of which were all identical in length to the ANHV prototype strain. Translated amino acid genome identity between the new virus and the ANHV prototype strain was 93.1% (L), 84.6% (M), 94.7% (N) and 89.0% (NSs) (Table 2). Amino acid sequence distance between the new virus and other phleboviruses was relatively high at 38.8%–68.5% (L), 51.2%–79.3% (M), 41.1%–64.2% (N) and 74.0%–84.0% (NSs) (Table 2), which was compatible with the relatively long branches segregating the ANHV‐related clade from other phleboviruses in phylogenetic analyses.

**Table 2:** Estimates of evolutionary divergence between PEHV, ANHV and other phleboviruses

Gene	Distance between ANHV‐related strains						Distance between PEHV and most closely related phleboviruses								
	PEHV versus ANHV						BUJV	MUNV	CANV	PTV	CHV	RVFV	SFSV	SFNV	PV
	Size	NT	AA	dS	dN	% dN	AA (%)								
L	6,291	20.5	6.9	893	146	2.3	38.8	39.8	42.7	41.0	40.7	40.4	44.2	45.2	68.5
M	4,080	25.5	15.4	455	213	5.2	55.4	56.6	52.4	53.6	51.2	53.4	55.0	54.6	79.3
N	738	19.9	5.3	99	13	1.7	41.1	42.3	46.1	41.6	51.2	44.4	48.9	53.9	64.2
NSs	750	18.3	11.0	86	32	4.3	74.0	77.5	78.0	81.5	78.5	77.5	80.5	84.0	84.0

Note

The number of amino acid or nucleic acid differences per site from between sequences is shown. Viruses Abbreviations: Anhanga (ANHV), Bujaru (BUJV), Munguba (MUNV), Candiru virus (CANV), Punta Toro (PTV), Chagres (CHV), Rift Valley fever (RVFV), Sandfly fever Sicilian (SFSV), Sandfly fever Naples (SFNV), Palma (PV), Penshurt (PEHV) viruses. dS, synonymous substitution, dN, non‐synonymous substitution.

So far, even the ANHV prototype strain is still unclassified by the International Committee on Taxonomy of Viruses (ICTV) and, since it could not be placed together with other viruses, neither based on serology (Nunes‐Neto et al., 2017) nor by molecular analyses, it may represent a new phlebovirus species. Considering that the recently proposed species demarcation criterion for phleboviruses is <95% amino acid identity within the translated L gene (Marklewitz, Palacios, Ebihara, Kuhn, & Junglen, 2019) and the 6.9% distance between the new ANHV‐related virus from Costa Rica and the Brazilian ANHV prototype strain in the translated L gene, these two viruses may correspond to two separate, yet monophyletic, species. In case the ICTV decides to classify the new virus from this study as a distinct species, we provisionally propose the name PEHV, according to the location of the sloth sanctuary.

Non‐synonymous mutations between PEHV and the genetically closely related ANHV prototype strain occurred significantly more frequently in M and NSs compared to L and N genes (chi‐square, *p* < .0001 for all comparisons; Table 2). The envelope glycoproteins Gn and Gc encoded by the M segment are associated with cellular attachment and viral entry and are targets for neutralizing antibodies (Venturi et al., 2007), and the NSs is associated with virulence and interferon antagonism in vertebrate cells (Billecocq et al., 2004). Therefore, the relatively higher divergence could be due to selective pressure associated with host adaptation and immune evasion during the genealogy of the two ANHV‐related viruses, that is the ANHV prototype strain and PEHV. However, mean dN/dS ratios ranged from 0.02 to 0.15 across all genes in a subset of phleboviruses genetically related to the ANHV‐related clade, irrespective of the method used (Figure 1d). Similarly, no sites showed significant evidence for positive selection. It should be noted that the apparent absence of positively selected sites might be biased by to insufficient number of sequences from individual viral species for robust formal selection pressure analyses. However, near‐complete absence of positively selected sites and similarly low mean dN/dS rates have been reported for larger phlebovirus data sets (Lam et al., 2013), suggesting robustness of our analyses. Significantly lower positive selection pressure in many arboviruses compared to other viruses is likely associated with their need to constantly adapt to both vertebrate and invertebrate hosts during their life cycle (Lequime, Fontaine, Ar Gouilh, Moltini‐Conclois, & Lambrechts, 2016; Sironi, Forni, Clerici, & Cagliani, 2016).

## 4 DISCUSSION

Hypothetically, one of the reasons for the lack of ANHV detection after the 1960s could be that the previous isolation was an accidental spillover infection into sloths from an unknown source. Here, we confirmed the association of ANHV‐related viruses with both extant two‐toed sloth species in the *Choloepus* genus across 3,000 km distance and half a century between the both detections. Future studies should address the identification of arthropod vectors involved in the life cycle of ANHV‐related viruses, as well as seroprevalence studies to ascertain their vertebrate hosts and zoonotic potential. Serological studies should ideally rely on both ANHV and PEHV isolates, since even the genetically closely related ANHV and PEHV may not cross‐react in serological assays, given the lack of cross‐reactivity between phleboviruses showing comparable genomic distances in their L and M genes (Marklewitz, Dutari, et al., 2019).

Finally, sloths have peculiar characteristics such as low body temperature and low metabolic rates (Cliffe et al., 2018; Pauli et al., 2014; Pauli, Peery, Fountain, & Karasov, 2016), likely associated with a low‐calorie diet that is high in toxicity and requires atypically long digestion periods (Cliffe et al., 2018). Even if the infected animal was apparently healthy, viral infection can affect the host metabolism on the cellular level and potentially also on the level of the whole organism (Byers, Fleshman, Perera, & Molins, 2019; Gonzalez Plaza, Hulak, Kausova, Zhumadilov, & Akilzhanova, 2016). Whether acute viral infections in general and phlebovirus infections in particular may thus be harmful to sloths due to their metabolic constraints remains an intriguing question.

## CONFLICT OF INTEREST

The authors declare no conflict of interest.

## ETHICAL APPROVAL

The authors confirm that the ethical policies of the journal, as noted on the journal's author guidelines page, have been adhered, and the appropriate ethical review committee approval has been received. The protocol and procedure for sampling the sloths were approved by the Costa Rican National Council in the Management of Biodiversity that regulates wild animal welfare (resolution R‐026‐OT‐CONAGEBIO). All sloth samples were taken by trained veterinarians in the sanctuary, collected as part of routine examinations according to national guidelines for animal care described in the Costa Rica National Law for Animal Welfare 7451.
